# T30. TIPPING POINTS – PREDICTING TRANSITIONS TO PSYCHOSIS IN AT-RISK YOUNG PEOPLE

**DOI:** 10.1093/schbul/sby016.306

**Published:** 2018-04-01

**Authors:** Jessica A Hartmann, Patrick D McGorry, Marieke Wichers, Barnaby Nelson

**Affiliations:** 1 Orygen, the National Centre of Excellence in Youth Mental Health; 2 University of Groningen, University Medical Center Groningen, Interdisciplinary Center Psychopathology and Emotion regulation

## Abstract

**Background:**

In traditional psychosis prediction research, the assumption is that a single “snapshot” of clinical disturbance at time point one (i.e. baseline) can reliably predict the future emergence of psychosis over time (i.e., follow-up). This is a linear, static approach to psychosis prediction. However, the field increasingly recognizes that mental health behaves as a non-linear, dynamic system, common to other complex structures such as ecosystems, financial markets or the climate.

**Methods:**

Increasing evidence points toward the existence of generic “tipping points” in these complex dynamic systems. A tipping point refers to a critical threshold whereby a system shifts from one state into another. Evidence suggests there are universal early warning signals/resilience indicators (such as a phenomenon called ‘critical slowing down’), which predict close proximity to a critical tipping point.

**Results:**

There is growing evidence for the presence of these early warning signals in psychopathology. This presentation will introduce theoretical concepts of tipping points and resilience indicators in the context of transitioning from at-risk mental state to frank psychosis.

**Discussion:**

This new framework may represent a paradigm shift from static prediction approaches to dynamic, individualized models of psychosis prediction and may inform the development of new clinical identification tools and early and individualized interventions to prevent such transitions.

